# Nocturnal Stage 1 Hypertension Defined by 2025 Guidelines in Adults With Chronic Kidney Disease

**DOI:** 10.1001/jamanetworkopen.2025.54035

**Published:** 2026-01-14

**Authors:** Ting Zhang, Zhengping Zhou, Qiong Li, Xiaoyu Cai, Lin Lin, Hui Peng, Man Li, Cheng Wang, Xinying Jiang

**Affiliations:** 1 Division of Nephrology, Department of Medicine, the Fifth Affiliated Hospital Sun Yat-sen University, Zhuhai, Guangdong, China; 2 Guangdong Provincial Engineering Research Center of Molecular Imaging, the Fifth Affiliated Hospital Sun Yat-sen University, Zhuhai, Guangdong, China; 3 Division of Nephrology, Department of Medicine, the Third Affiliated Hospital Sun Yat-sen University, Guangzhou, Guangdong, China

## Abstract

**Importance:**

The 2025 American College of Cardiology/American Heart Association (ACC/AHA) blood pressure (BP) guideline introduced new hypertension criteria. However, the association between newly defined nocturnal stage 1 hypertension (nocturnal BP 110-120/65-70 mm Hg) and kidney function progression in the population not receiving dialysis with chronic kidney disease (CKD) in China remains unclear.

**Objective:**

To determine whether nocturnal stage 1 hypertension as defined by 2025 ACC/AHA guidelines in patients with CKD is associated with kidney function progression.

**Design, Setting, and Participants:**

A retrospective cohort study involving patients with CKD not receiving dialysis was conducted in China at the Third Affiliated Hospital of Sun Yat-sen University from August 2010 to December 2017 and at the Fifth Affiliated Hospital of Sun Yat-sen University from November 2017 to December 2024.

**Exposure:**

Participants were categorized into 3 groups based on nocturnal BP measurements according to the 2025 ACC/AHA guidelines: nonhypertension (nocturnal BP <110/65 mm Hg), stage 1 hypertension (nocturnal BP 110-120/65-70 mm Hg), and stage 2 hypertension (nocturnal BP ≥120/70 mm Hg).

**Main Outcomes and Measures:**

The primary outcome was a composite of kidney failure requiring replacement therapy (KFRT) and worsening kidney function (WKF). Secondary outcomes included the individual components of the primary outcome. Cox regression models were employed to assess the association between nocturnal stage 1 hypertension and outcomes.

**Results:**

Among 2418 participants with CKD stage 1 through 4 not receiving dialysis included in the study, 1412 (58.4%) were men, the mean (SD) age was 45.4 (14.4) years, 436 (18.0%) had nocturnal nonhypertension, 345 (14.3%) had nocturnal stage 1 hypertension, and 1637 (67.7%) had nocturnal stage 2 hypertension. Over a median (IQR) follow-up of 3.9 (1.5-4.7) years, 394 composite kidney outcomes, 203 cases of KFRT, and 235 instances of WKF were recorded. After adjusting for confounders, nocturnal stage 1 hypertension (compared with nocturnal nonhypertension) was independently associated with an increased risk of KFRT (hazard ratio [HR], 2.37; 95% CI, 1.17-4.82), WKF (HR, 3.79; 95% CI, 1.04-13.84), and the composite kidney outcome (HR, 2.49; 95% CI, 1.31-4.72). In the older population (aged ≥65 years), nocturnal BP less than 110/65 mm Hg was associated with a higher risk of WKF and the composite kidney outcome compared with nocturnal BP in the range of 110/65 to 120/70 mm Hg.

**Conclusions and Relevance:**

In this cohort study of adults with CKD not receiving dialysis, nocturnal stage 1 hypertension as defined by the 2025 ACC/AHA guidelines was independently associated with an increased risk of CKD progression.

## Key Points

**Question:**

Is nocturnal stage 1 hypertension as defined by 2025 American College of Cardiology/American Heart Association (ACC/AHA) guidelines in patients with chronic kidney disease (CKD) associated with decline in kidney function?

**Findings:**

In this cohort study of 2418 patients with CKD not receiving dialysis, nocturnal stage 1 hypertension, as defined by the 2025 ACC/AHA guidelines, was independently associated with kidney function deterioration.

**Meaning:**

These findings suggest that an optimal management strategy is needed for patients with CKD and stage 1 hypertension who are not receiving dialysis to benefit kidney outcomes.

## Teaser

This cohort study evaluates whether nocturnal stage 1 hypertension as defined by 2025 American College of Cardiology/American Heart Association guidelines in patients with chronic kidney disease (CKD) is associated with worsening of kidney disease.

## Introduction

Chronic kidney disease (CKD) represents a significant global public health challenge, characterized by its high prevalence, low awareness, substantial health care costs, and poor clinical outcomes.^1^ The Sixth China Chronic Disease and Risk Factor Surveillance reported that, in 2018 to 2019, an estimated 82 million adults in China were affected by CKD.^2^ Hypertension is both a cause and a consequence of CKD, with their coexistence linked to an elevated risk of cardiocerebrovascular (CVD) and kidney events.^3,4^ Consequently, effective blood pressure (BP) management is a critical strategy in CKD care.

Ambulatory BP monitoring (ABPM), which provides continuous 24-hour BP recordings, is crucial for identifying BP phenotypes and is considered a robust prognostic tool for patients with CKD compared with office BP measurements.^5^ The Kidney Disease: Improving Global Outcomes (KDIGO) guidelines currently recommend ABPM as a complement to office BP for hypertension management in CKD.^6^ Numerous studies have demonstrated that ABP, particularly nocturnal BP, offers a more accurate assessment of the risk for hypertension-related adverse outcomes than office BP.^7,8^ Additionally, patients with CKD are more likely to experience nocturnal hypertension than the general population due to factors such as increased salt sensitivity, sympathetic hyperactivity, and activation of the renin-angiotensin-aldosterone system.^9^ Thus, nocturnal BP warrants heightened attention in CKD management.

The 2017 American College of Cardiology/American Heart Association (ACC/AHA) BP guidelines revised the definition of hypertension, lowering the threshold for office systolic BP (SBP) and diastolic BP (DBP) from 140/90 mm Hg to 130/80 mm Hg.^6,10^ This definition was maintained in recently announced 2025 ACC/AHA BP guidelines.^11^ Similarly, the nocturnal hypertension threshold was reduced from 120/70 mm Hg to 110/65 mm Hg. A nationwide survey in China showed that the prevalence of hypertension based on the 2025 ACC/AHA guideline (46.4%) was twice as high as that based on Chinese hypertension guidelines (23.2%).^12^ An increase in the prevalence of hypertension under the new definition would lead to considerable growth of clinical and economic burden.^13^ According to the 2025 ACC/AHA guidelines, for patients with BP of 130-139/80-89 mm Hg, only those with high risk factors would require treatment.^11^ Considering the overall low awareness of hypertension in China, the increase in the number of treated hypertensive patients would be negligible.^14^ Therefore, the application of a new hypertension definition could burden patients and society and may confer limited clinical benefit in the general population in China. However, accounting for the high prevalence of hypertension in the population with CKD, it is worthwhile to explore the clinical value of a new hypertension definition in CKD, particularly for nocturnal hypertension. Despite China having the largest population of patients with CKD globally, research on the implications of these newly defined hypertension criteria in Chinese populations with CKD, particularly nocturnal hypertension, remains limited.^15^ This study aims to assess the clinical value of the newly defined nocturnal hypertension (nocturnal stage 1 hypertension) as per the 2025 ACC/AHA guidelines in a CKD cohort not receiving dialysis.

## Methods

This study was reported as per Strengthening the Reporting of Observational Studies in Epidemiology (STROBE) reporting guidelines. Ethical approval was obtained from the ethics committee and the institutional review board of Third and Fifth Affiliated Hospitals of Sun Yat-sen University. Written informed consent was obtained from all participants.

### Study Population

This retrospective cohort study was carried out independently at 2 university teaching hospitals in Guangdong, China. Patients were enrolled from August 2010, and follow-up ended in December 2017 at the Third Affiliated Hospital of Sun Yat-sen University. From November 2017 to July 2021, patients were enrolled at the Fifth Affiliated Hospital of Sun Yat-sen University, and follow-up was terminated in December 2024.

The inclusion criteria were age between 18 and 75 years old and diagnosis of CKD at stage 1 to stage 4 at study enrollment. The exclusion criteria were as follows: (1) inadequate ambulatory BP readings; (2) malignant neoplasm; (3) medical history of atrial fibrillation; (4) acute kidney injury defined as a 30% or more decrease in estimated glomerular filtration rate (eGFR) within 3 months; (5) acute infection; (6) current pregnancy or lactation; (7) ongoing medium to high dose glucocorticoid or immunosuppressant (cyclosporine or tacrolimus) treatment; or (8) any acute CVD in the previous 3 months. Cases for which a minimum of 6-month follow-up could not be obtained were further excluded (2 deceased patients and 23 lost to follow-up). See eFigure 1 in Supplement 1 for study enrollment details.

### BP Measurements

Office BP was measured 3 times using a validated oscillometric device in the sitting position after a 5-minute rest with 1- to 2-minute intervals between measurements. The mean of 3 consecutive measurements was used in the analyses.

The ABPM was conducted at the time of first admission to our hospitals with validated automatic devices, and the time 0 for survival analyses was defined as the date of this measurement. Ambulatory BP was obtained every 15 minutes during the day and every 30 minutes during the night. Daytime and nighttime periods were defined according to patients’ daily diaries. With at least 20 valid readings during daytime and 7 valid readings during nighttime, a minimum of 70% of BP records were needed for a valid ABPM measurement.

### Kidney Function Assessment and Kidney Outcomes

Serum creatinine was measured using an isotope dilution mass spectrometry–traceable creatinine assay, and eGFR was calculated through the CKD-Epidemiology Collaboration (CKD-EPI) formula. CKD stage was defined using the KDIGO definition. The primary study end point was a composite outcome of kidney function defined as the occurrence of 30% or more sustained decline in eGFR, doubling of serum creatinine, initiation of dialysis, or kidney transplantation, whichever occurred first. Secondary end points were kidney failure requiring replacement therapy (KFRT) (dialysis or transplantation) and worsening kidney function (WKF) (eGFR decline ≥30% or doubling of serum creatinine). Follow-up was performed every 6 months by telephone interviews or routine clinical visits until patient death, loss to follow-up, or the attainment of the predetermined follow-up cutoff date.

### Definitions of Hypertension

History of hypertension was defined as an office BP above 140/90 mm Hg, a previous history of hypertension, or use of antihypertensive medication based on the 2024 Chinese Guidelines for the Prevention and Treatment of Hypertension.^16^ Nocturnal BP was categorized according to the 2025 ACC/AHA BP guidelines as follows: normal nocturnal BP of SBP lower than 100 mm Hg and DBP lower than 65 mm Hg, elevated nocturnal BP of SBP 100 to 110 mm Hg and DBP lower than 65 mm Hg, stage 1 nocturnal hypertension of SBP 110 to 120 mm Hg or DBP 65 to 70 mm Hg, and stage 2 nocturnal hypertension of SBP 120 mm Hg and higher or DBP 70 mm Hg and higher. In the analysis, normal and elevated nocturnal BP were combined and defined as nocturnal nonhypertension. Participants with nocturnal hypertension were further grouped as follows: nocturnal isolated systolic hypertension (NISH) with nocturnal SBP 110 mm Hg and higher and DBP lower than 65 mm Hg, nocturnal isolated diastolic hypertension (NIDH) with nocturnal SBP lower than 110 mm Hg and DBP 65 mm Hg and higher, and nocturnal systolic-diastolic hypertension (NSDH) with nocturnal SBP 110 mm Hg and higher and DBP 65 mm Hg and higher.

### Statistical Analysis

All data analyses were performed using R version 4.4.2 (R Project for Statistical Analysis). The Shapiro-Wilk normality test was used to test normality. Data were reported as the mean (SD) for normal continuous variables, median (IQR) for nonnormal continuous variables, and frequency (percentage) for discrete variables. Differences among groups were compared using analysis of variance for normally distributed data, Kruskal-Wallis test for nonnormally distributed data, and χ^2^ test for discrete variables. The percentage of missing values ranged from less than 5% for fasting plasma glucose to 6% for urine protein-to-creatinine ratio (UPCR). Incomplete variables were imputed through multiple imputation by chained equations with 5 imputations. The age- and sex-adjusted incidence rates per 1000 person-years of study outcomes were calculated using the direct method. We conducted Cox proportional hazards regression analysis to evaluate the association of stage 1 hypertension with kidney disease progression. Model 1 was adjusted for sex, age, and body mass index (BMI). Model 2 was adjusted for sex, age, BMI, cigarette smoking, diabetes, CVD history, antihypertensive drugs, hemoglobin, albumin, high-density lipoprotein cholesterol (HDL-C), serum creatinine, and UPCR. Model 3 was adjusted for model 2 covariates with the addition of daytime SBP. Kaplan-Meier survival curves with a log-rank test were plotted.

We performed 6 sensitivity analyses to confirm whether the result was robust. First, nocturnal SBP and DBP were explored as continuous variables for potential nonlinear association with the risk of kidney function decline using restricted cubic splines with 4 knots. Second, we estimated the cumulative incidence of kidney outcomes in model 3 by treating death as a competing risk in terms of time from 6-year survival. We further calculated subhazard ratios (sHR) using the Fine-Gray competing risk regression analysis. Third, to reduce potential selection bias, we generated 2 matched cohorts (stage 1 hypertension vs nonhypertension and stage 2 hypertension vs nonhypertension) by 1:1 propensity score matching using the nearest neighbor method without replacement. Propensity scores were estimated by logistic regression on the basis of covariates including sex, age, BMI, cigarette smoking, diabetes, CVD history, antihypertensive drugs, hemoglobin, albumin, HDL-C, serum creatinine, and UPCR. Fourth, in subgroups of older patients (aged ≥65 years) and younger patients (aged <65 years), stage 1 hypertension was set as the reference in Cox proportional hazards regression. Fifth, keeping in view the increasing incidence of diastolic hypertension under the 2025 ACC/AHA guideline, investigating the association of NIDH with adverse outcomes was an absolute requirement. Sixth, stratified and interaction analyses were also performed to verify the association between nocturnal stage 1 hypertension and kidney outcomes. All significance tests were 2-sided with significance levels of .05.

## Results

### Baseline Characteristics

A total of 2418 patients with CKD stages 1 through 4 not receiving dialysis were included in the study (Table). The mean (SD) age of the participants was 45 (14) years, and 1412 (58.4%) were male. The majority of patients had CKD stage 1 (925 patients [38.3%]), followed by 600 with stage 2 (24.8%) and 592 with stage 3 (24.5%), with a smaller proportion in stage 4 (301 patients [12.4%]). According to the 2024 Chinese Guidelines for the Prevention and Treatment of Hypertension, 1443 of the overall participants (59.7%) were classified as hypertensive. The study population was further divided into 3 groups: 436 participants (18.0%) had a nocturnal BP of 110/65 mm Hg or lower (nonhypertension group), 345 (14.3%) had nocturnal BP 110-120/65-70 mm Hg (stage 1 hypertension group), and 1637 (67.7%) had a nocturnal BP of 120/70 mm Hg or higher (stage 2 hypertension group), based on the 2025 ACC/AHA guidelines. Under the new guidelines, there were 1982 participants (82.0%) with nocturnal hypertension. As BP increased, there was a higher prevalence of cigarette smoking, diabetes, CVD history, and antihypertensive drug use.

**Table.** Baseline Characteristics of Study Participants

Characteristic	Participants, No. (%)				*P* value
	Overall (N = 2418)	Nonhypertension (n = 436)	Stage 1 hypertension (n = 345)	Stage 2 hypertension (n = 1637)	
Clinic SBP, mmHg	135.8 (23.1)	119.5 (17.2)	127.7 (18.1)	141.9 (22.8)	<.001
Clinic DBP, mmHg	85.6 (14.3)	76.1 (10.4)	80.3 (11.3)	89.3 (14.2)	<.001
24 h mean SBP, mmHg	125.2 (16.1)	107.8 (8.0)	115.9 (8.4)	131.7 (14.5)	<.001
24 h mean DBP, mmHg	79.8 (11.2)	66.3 (5.5)	72.6 (5.0)	84.8 (9.3)	<.001
Daytime SBP, mmHg	126.6 (16.1)	110.2 (8.8)	118.0 (9.4)	132.8 (14.8)	<.001
Daytime DBP, mmHg	81.0 (11.3)	68.2 (6.2)	74.3 (6.0)	85.8 (9.7)	<.001
Nocturnal SBP, mmHg	119.6 (17.9)	98.2 (6.5)	108.0 (6.2)	127.7 (15.4)	<.001
Nocturnal DBP, mmHg	75.0 (12.5)	58.6 (4.3)	66.1 (2.8)	81.3 (9.7)	<.001
Age, y	45.4 (14.4)	37.7 (13.6)	42.3 (15.0)	48.2 (13.5)	<.001
Sex					
Female	1006 (41.6)	252 (57.8)	150 (43.5)	604 (36.9)	<.001
Male	1412 (58.4)	184 (42.2)	195 (56.5)	1033 (63.1)	
BMI^a^	24.1 (3.8)	22.3 (3.7)	23.6 (3.73)	24.6 (3.8)	<.001
Cigarette smoking	654 (27.0)	76 (17.4)	66 (19.1)	512 (31.3)	<.001
Diabetes	467 (19.3)	28 (6.42)	42 (12.2)	397 (24.3)	<.001
CVD history	191 (7.9)	7 (1.6)	16 (4.6)	168 (10.3)	<.001
Hypertension history	1443 (59.7)	89 (20.4)	134 (38.8)	1220 (74.5)	<.001
Antihypertensive drugs	1645 (68.0)	218 (50.0)	199 (57.7)	1228 (75.0)	<.001
Serum creatinine, mg/dL	1.1 (0.8-1.6)	0.8 (0.7-1.1)	1.0 (0.7-1.2)	1.2 (0.9-1.8)	<.001
eGFR, ml/min/1.73m^2^	76.7 (46.4-103.3)	101.0 (74.6-118.3)	90.6 (64.3-108.6)	63.9 (38.7-95.4)	<.001
UPCR, mg/g	350.5 (77.1-1234.1)	128.4 (37.4-594.7)	196.6 (65.0-670.5)	451.8 (107.0-1828.8)	<.001
FBG, mg/dL	86.5 (79.3-99.1)	84.7 (77.5-91.9)	86.5 (79.3-93.7)	88.3 (79.3-102.7)	<.001
Hemoglobin, g/dL	13.1 (11.5-14.5)	13.0 (11.7-14.3)	13.3 (12.0-14.8)	13.0 (11.3-14.6)	.02
Albumin, g/dL	3.7 (0.8)	3.7 (0.9)	3.7 (0.9)	3.7 (0.8)	.36
LDL-C, mg/dL	112.1 (88.9-150.8)	108.3 (85.1-150.8)	112.1 (88.9-146.9)	112.1 (88.9-150.8)	.61
HDL-C, mg/dL	42.5 (34.8-54.1)	46.4 (38.7-58.0)	46.4 (38.7-54.1)	42.5 (34.8-50.3)	<.001
Triglyceride, mg/dL	132.8 (97.4-203.6)	106.2 (70.8-159.3)	123.9 (88.5-185.9)	141.6 (97.4-212.4)	<.001
Total cholesterol, mg/dL	189.5 (158.5-235.9)	181.7 (150.8-232.0)	189.5 (154.7-235.9)	189.5 (158.5-239.7)	.20

Abbreviations: BMI, body mass index, calculated as weight in kilograms divided by height in meters squared; CVD, cardiocerebrovascular disease; DBP, diastolic blood pressure; eGFR, estimated glomerular filtration rate; FBG, fasting blood glucose; HDL-C, high-density lipoprotein cholesterol; LDL-C, low-density lipoprotein cholesterol; SBP, systolic blood pressure; UPCR, urine protein-to-creatinine ratio.

SI conversion factors: To convert albumin to grams per liter, multiply by 10.0; creatinine to micromoles per liter, multiply by 88.4; glucose to millimoles per liter, multiply by 0.0555; hemoglobin to grams per liter, multiply by 10.0; high-density lipoprotein cholesterol to millimoles per liter, multiply by 0.259; low-density lipoprotein cholesterol to millimoles per liter, multiply by 0.259; total cholesterol to millimoles per liter, multiply by 0.259; triglycerides to millimoles per liter, multiply by 0.113.

### Incidence of Kidney Outcomes

**Figure 1.**
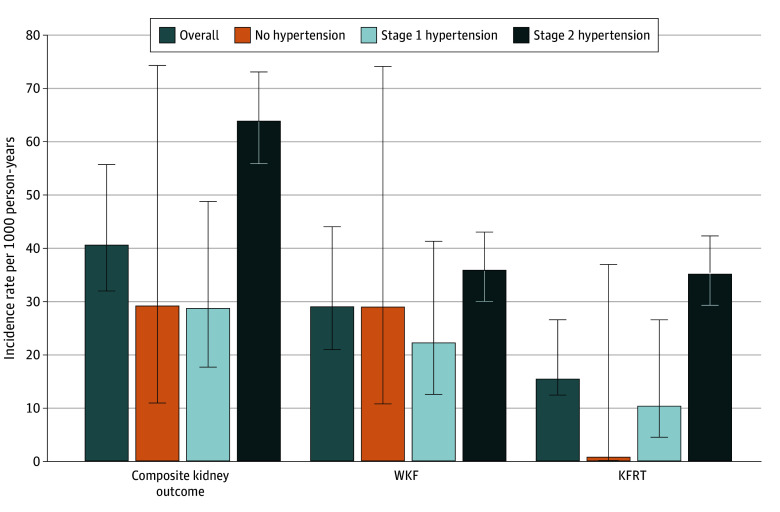
Age- and Sex- Adjusted Incidence Rates (Per 1000 Person-Years) for Primary and Secondary Outcomes Error bars represent 95% CIs. WKF indicates worsening kidney function; KFRT, kidney failure requiring replacement therapy.

There were 353 participants lost to follow-up, and the follow-up rate was 85.4%. The results are presented as age- and sex-adjusted incidence rates with corresponding 95% CIs per 1000 person-years over a median (IQR) follow-up of 3.9 (1.5-4.7) years (Figure 1). A total of 394 composite kidney events, 235 cases of WKF, and 203 instances of KFRT occurred. The incidence rates for the primary outcome were 29.1, 28.6, and 63.7 per 1000 person-years in the nonhypertension, stage 1 hypertension, and stage 2 hypertension groups, respectively. The highest incidence rates for WKF and KFRT were observed in the stage 2 hypertension group (35.8 and 35.0 per 1000 person-years, respectively).

### Association of Nocturnal Stage 1 Hypertension With Kidney Outcomes

**Figure 2.**
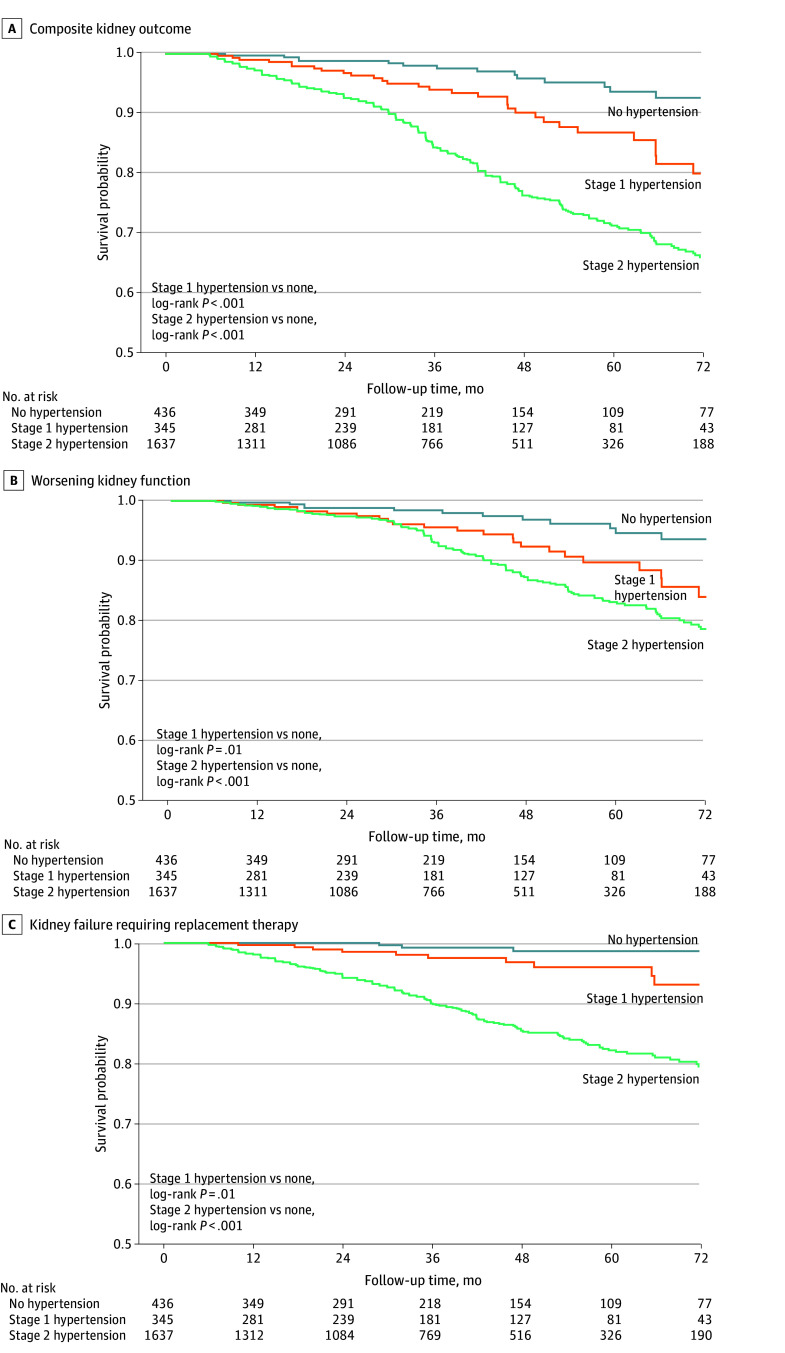
Kaplan-Meier Survival Curves for Kidney Outcomes by Nocturnal Hypertension Status

**Figure 3.**
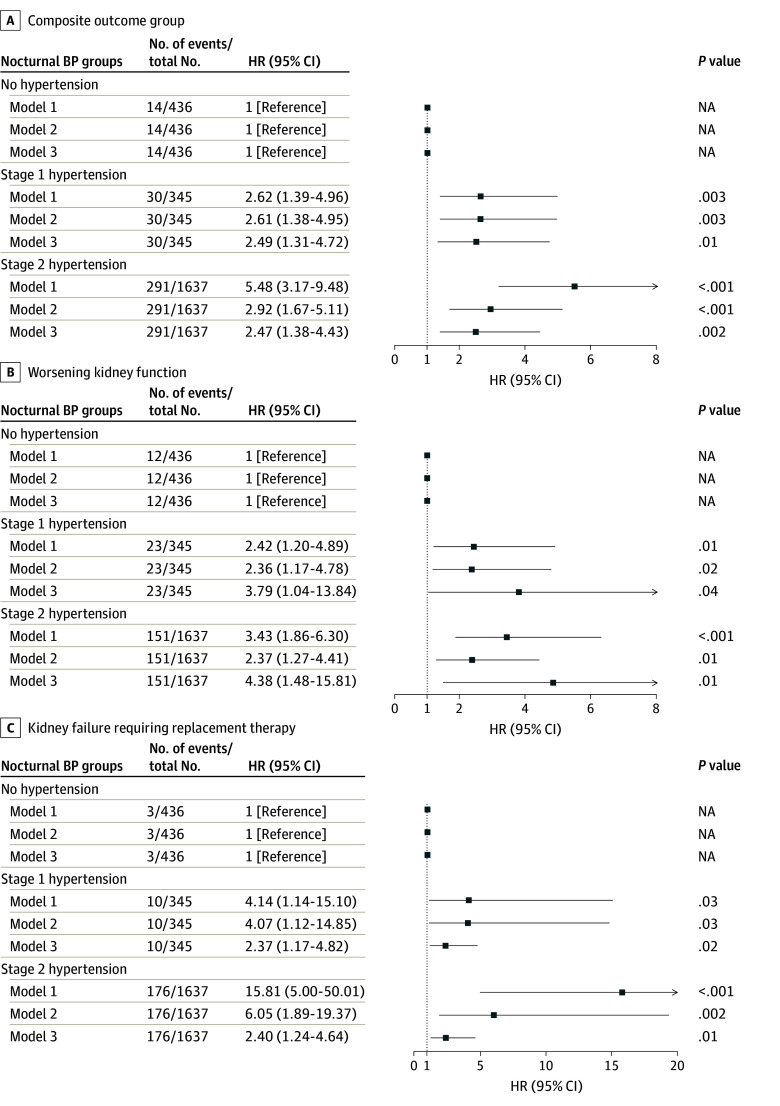
Multivariable Cox Analyses of Association Between Nocturnal Hypertension Status and Kidney Outcomes Model 1 adjusted for age, sex, body mass index; model 2 adjusted for model 1 plus cigarette smoking, diabetes, cardiocerebrovascular disease history, antihypertensive drugs, hemoglobin, albumin, high-density lipoprotein cholesterol, serum creatinine, and urine protein-to-creatinine ratio; model 3 adjusted for model 2 plus daytime SBP. BP indicates blood pressure; HR, hazard ratio.

Kaplan-Meier survival curves revealed significant differences in survival probability across the nocturnal hypertension groups (Figure 2). The survival probability for both primary and secondary outcomes was lowest in the stage 2 hypertension group, followed by the stage 1 hypertension group. All multivariable Cox proportional hazards regression models met the proportional hazards assumption. Nocturnal stage 1 hypertension (vs nocturnal nonhypertension) was associated with an increased risk for the primary outcome (HR, 2.49; 95% CI, 1.31-4.72), WKF (HR, 3.79; 95% CI, 1.04-13.84), and KFRT (HR, 2.37; 95% CI, 1.17-4.82) in model 3 (Figure 3).

### Propensity Score Matching

Propensity score matching resulted in 2 cohorts of 292 patients in the nonhypertension or stage 1 hypertension group and 363 patients in the nonhypertension or stage 2 hypertension group, matched at a 1:1 ratio. The relative density of propensity scores and standardized differences before and after matching are shown in eFigure 2 in Supplement 1, indicating balance between the compared cohorts. After matching, patient characteristics were balanced, with no statistically significant difference across all variables (eTable 1 in Supplement 1). Furthermore, Kaplan-Meier analysis demonstrated a significant effect of both stage 1 (χ^2^_1_ = 4.61; log-rank *P* = .03) and stage 2 (χ^2^_1_ = 6.32; log-rank *P* = .01) nocturnal hypertension on the composite kidney outcome after matching. In Cox regression adjusted for daytime SBP, stage 1 nocturnal hypertension remained a significant factor associated with risk of the composite kidney outcome (HR, 2.10; 95% CI, 1.03-4.30).

### Sensitivity Analyses

Restricted cubic spline (RCS) analysis revealed a clear nonlinear association between nocturnal SBP (χ^2^_2_ = 7.9; nonlinear *P* = .02) or DBP (χ^2^_2_ = 12.7; nonlinear *P* < .001) and the composite kidney outcomes (eFigure 3 in Supplement 1). The RCS results indicated cutoff points for nocturnal SBP and DBP at 106 mm Hg and 57 mm Hg, respectively. When nocturnal SBP and DBP were taken as continuous variables, both were not independently associated with composite kidney outcomes, WKF, or KFRT (eTable 2 in Supplement 1). sHRs were estimated using competing risk Cox regression with death as a competing risk and composite kidney outcome as the outcome. In the fully adjusted model, sHRs remained statistically significant for the composite kidney outcome (stage 1 vs nonhypertension: sHR, 2.52; 95% CI, 1.35-4.69). Gray tests further showed a significantly higher incidence of composite kidney outcome in the stage 1 hypertension group compared with the nonhypertension group (eFigure 4 in Supplement 1). Considering the potential influence of age, in individuals aged 65 years or older, nocturnal nonhypertension emerged as a factor associated with risk for the composite kidney outcome (HR, 5.50; 95% CI, 1.05-28.71; *P* = .04) and WKF (HR, 5.65; 95% CI, 1.02-31.32; *P* = .05) when nocturnal stage 1 hypertension served as the reference (eTable 3 in Supplement 1). This study further examined the separate and combined outcomes of nocturnal SBP and DBP. The study population was further divided into three 3 groups: 323 participants (13.4%) had NIDH, 66 (2.7%) had NISH, and 1593 (65.9%) had NSDH. In multivariable models adjusted for sex, age, and BMI, the HRs for the composite kidney outcome were 2.88 for NIDH, 3.34 for NISH, and 5.61 for NSDH. In the fully adjusted model, the association between NIDH or NSDH and the composite kidney outcome was partially attenuated but remained statistically significant (eFigure 5 in Supplement 1). Results for WKF and KFRT were less precise due to wider CIs and smaller sample sizes. Subgroup analyses revealed no significant interactions across subgroups. The association between nocturnal stage 1 hypertension and the composite kidney outcome remained significant in subgroups of female patients, individuals with higher eGFR, and those without a history of CVD, hypertension, proteinuria, diabetes or antihypertensive drug use after full adjustment. The lack of statistical significance in other subgroup analyses is likely due to the reduced sample size within each subgroup after stratification (eFigure 6 in Supplement 1).

## Discussion

To our knowledge, this cohort study is the first to examine the association between nocturnal stage 1 hypertension and kidney function progression in patients with CKD not receiving dialysis. The findings suggested that nocturnal stage 1 hypertension, as defined by the 2025 ACC/AHA guidelines, was associated with increased risk of kidney function deterioration, particularly in younger patients, compared with nocturnal nonhypertension.

In this study, the prevalence of office hypertension was 59.7% according to the Chinese guidelines, aligning with other epidemiological studies conducted in the Chinese population with CKD.^2^ To evaluate the applicability of the 2025 ACC/AHA guidelines in the Chinese population with CKD not receiving dialysis, a national multicenter cross-sectional study^17^ revealed that the prevalence of hypertension based on the new, more stringent BP categories was 79.8%, significantly higher than the 67.3% estimated using the Chinese guideline’s definition. The present study further reported that 82.0% of participants had nocturnal hypertension, including 67.7% with stage 2 hypertension as previously defined and 14.3% with stage 1 hypertension, according to the 2025 ACC/AHA guidelines. Similarly, the incidence of nocturnal stage 1 hypertension in the general Colombian population was 24% when applying the ACC/AHA criteria.^18^ To date, no studies have reported the prevalence of the newly defined nocturnal stage 1 hypertension in populations with CKD. The implementation of the 2025 ACC/AHA guidelines has led to a significant increase in hypertension prevalence, contributing to both psychological and economic burdens on patients with CKD.

In addition to monitoring the rising incidence of newly defined hypertension, it is crucial to investigate the potential impact of this BP level on prognosis. Since the release of the 2025 ACC/AHA guidelines, multiple studies have examined the prognostic significance of office-measured stage 1 hypertension for cardiovascular events and mortality.^19,20,21,22,23,24,25^ However, limited research exists on the prognostic value of stage 1 hypertension concerning kidney outcomes. Several studies in the Asian population have shown that stage 1 hypertension is associated with a higher risk of proteinuria and kidney function deterioration.^26,27,28^ Notably, these studies excluded patients with prior kidney or cardiovascular disease, and evidence within populations with CKD remains scarce. One observational study in young adults with CKD found that an SBP of 130 mm Hg or higher was significantly associated with a higher risk of CVD events and CKD progression compared with an SBP lower than 120 mm Hg.^29^ Furthermore, there is a lack of high-quality evidence supporting the benefits of intensive antihypertensive treatment for improving kidney prognosis. Three large RCTs in populations with CKD showed that intensified BP lowering did not provide additional benefits for kidney outcomes.^30,31,32^ Even in the Systolic Blood Pressure Intervention Trial study, participants with CKD assigned to intensive BP lowering (office SBP <120 mm Hg) exhibited a reduced rate of CVD events and mortality but no effect on kidney outcomes.^33^ Overall, current RCTs do not support intensive BP control for improving kidney outcomes. Consequently, the KDIGO 2021 Clinical Practice Guideline for the Management of BP in CKD provides a weak recommendation for targeting an SBP less than 120 mm Hg, according to Grading of Recommendations Assessment, Development, and Evaluation standards.^6^

Nocturnal hypertension and disruptions in BP circadian rhythms are prevalent among patients with CKD. Previous studies have consistently demonstrated that nocturnal stage 2 hypertension (nocturnal BP ≥120/70 mm Hg) is associated with increased risk of CKD progression, independent of office BP measurements.^8,34,35,36,37^ However, the association between nocturnal stage 1 hypertension and CKD progression has remained unclear. Despite the KDIGO 2021 Clinical Practice Guideline for the Management of BP in CKD recommending a target office SBP less than 120 mm Hg for adults with CKD and hypertension, the optimal target for nocturnal SBP, specifically whether it should be reduced to a corresponding threshold (nocturnal SBP<100 mm Hg), is yet to be determined.^6^ In the present study, nocturnal stage 1 hypertension, independent of daytime SBP, was associated with a higher risk of CKD progression (HR, 3.79; 95% CI, 1.04-13.84) and the initiation of kidney replacement therapy (HR, 2.37; 95% CI, 1.17-4.82). These results remained consistent after adjusting for competing risks and potential confounders. The RCS model further revealed that nocturnal SBP of 106 mm Hg and DBP of 57 mm Hg served as thresholds for estimating the composite kidney outcome in patients with CKD not receiving dialysis, aligning closely with the threshold of 110/65 recommended by the 2025 ACC/AHA guidelines for defining nocturnal hypertension.^38^ A recent study in China categorized patients with CKD into 4 quartiles based on nocturnal SBP, and the second quartile (110-124 mm Hg) was associated with kidney prognosis, with an HR of 1.975 (95% CI, 1.311-2.976).^37^ Additionally, a cross-sectional study from the same team divided nocturnal SBP into tertiles and found that tertile 3 (≥114 mm Hg) was linked to target organ damage in CKD.^39^ Our findings are consistent with these previous results.

In contrast, the findings in older adults were notably different. When the nocturnal stage 1 hypertension group (nocturnal BP 110-120/65-70 mm Hg) was used as a reference, the nocturnal nonhypertension group (nocturnal BP <110/65 mm Hg) was associated with a higher risk of composite kidney outcome among patients aged ≥65 years, but with opposite associations among patients aged younger than 65 years. This suggests that excessively low nocturnal BP may be detrimental and increase the risk of adverse events in older patients with CKD. The risk-benefit balance of intensive BP-lowering strategies, particularly in older patients, remain unclear. Intensive BP reduction is known to be associated with serious adverse events, such as acute kidney injury, hyperkalemia, and hypotension, especially in older and frail populations.^40^ A large study involving 158 713 older adults aged older than 60 years with CKD found that office SBP lower than 120 mm Hg was associated with increased mortality.^41^ Similarly, an RCT in the older Chinese population showed that intensive therapy targeting an SBP of 110 to 130 mm Hg reduced cardiovascular event risk but had no significant impact on kidney outcomes when compared with conventional therapy.^42^ Regarding nocturnal BP, no interventional data have been available for patients with CKD to date. Further high-quality studies with larger sample sizes are necessary to confirm these findings.

The diastolic threshold for hypertension proposed by the 2025 ACC/AHA guidelines was based on expert consensus rather than high-quality randomized clinical trials.^38^ An epidemiological study^43^ in Brazil reported a high prevalence of isolated diastolic hypertension (IDH) (85%) and a low prevalence of isolated systolic hypertension (ISH) (1.1%) in the newly defined stage 1 hypertension population, suggesting that the 2025 ACC/AHA guidelines may place undue emphasis on the diastolic component, while downplaying the significance of the systolic component in hypertension diagnosis. In the present study, the incidence of NIDH, NISH, and NSDH in the nocturnal hypertension group was 13.4%, 2.7%, and 65.9%, respectively. Although these proportions vary, IDH was also prevalent in other studies.^28,44,45^ Notably, a favorable value of NIDH was observed for composite kidney outcome and WKF, which aligns with findings from several studies.^28,44^ A cohort study^28^ in Korea with 3 030 884 participants found that both stage 1 and stage 2 IDH, as defined by the 2025 ACC/AHA guidelines, were associated with incident CKD, though this study focused on office BP rather than nocturnal BP. Another study in Taiwan, involving 284 597 participants, reported that both stage 1 and stage 2 office IDH, defined by the 2025 ACC/AHA guidelines, were linked to a higher risk of cardiovascular mortality but not all-natural mortality.^44^ However, some studies report conflicting results on this issue. In contrast to our findings, a study from the Chinese Cohort Study of Chronic Kidney Disease, which included 2024 patients with CKD (stages 1-4) found no association between isolated nocturnal diastolic hypertension (defined as nocturnal SBP <120 mm Hg and DBP ≥70 mm Hg) and kidney failure.^46^ Another study^45^ combining cross-sectional and longitudinal cohorts found no significant association between office IDH and cardiovascular outcomes, regardless of the definition applied. The discrepancies in BP measurement techniques, BP cutoff values, and study populations make direct comparisons with previous studies challenging.

### Limitations

The limitations of this study include its observational nature and focusing on a Chinese population, which limits the generalizability of the findings to other ethnic groups. Additionally, participants were recruited from 2 nephrology wards, representing a population with CKD not receiving dialysis, so the results may not be directly applicable to patients with end-stage kidney disease. Furthermore, we did not have repeated measures of ABPM and some laboratory indices in most of the participants during follow-up to elucidate time-dependent outcomes. Another deficiency of this study is that the sample size was relatively small, especially for the subgroup analysis, which results in a possibility of statistical deviation. In addition, we may have inadequately controlled for potential confounders due to the small sample size. Larger prospective cohort studies that encompass diverse ethnic populations are needed to provide more robust, evidence-based support.

## Conclusions

In this cohort study of Chinese adults with CKD not receiving dialysis, those with nocturnal stage 1 hypertension faced a higher risk of composite kidney outcomes compared with those with nocturnal nonhypertension. Due to the relatively insufficient sample size, the clinical impact of nocturnal stage 1 hypertension in older populations is still open to question.

## Notes

Supplement 1. eTable 1. Baseline Characteristics in the Matched Cohorts eTable 2. The Association Between Blood Pressure as Continuous Variables and Primary and Secondary Outcomes eTable 3. Association Between Nocturnal Hypertension and Composite Renal Outcome Among Younger and Older Groups eFigure 1. Flow Chart eFigure 2. Propensity Score Matching for Stage 1 vs Nonhypertension, Stage 2 vs Nonhypertension Cohorts eFigure 3. Restricted Cubic Spline Curves of Nocturnal SBP/DBP With Composite Renal Outcome eFigure 4. Competing Risk Model With Mortality as the Competing Risk, Showing Risk of Composite Renal Outcome With Stage 1 Hypertension eFigure 5. Association Between Nocturnal Blood Pressure Phenotype With (A) Composite Kidney Outcome, (B) WRF and (C) KFRT eFigure 6. Forest Plots for the Subgroup Analyses of the Association Between Nocturnal BP Categories and the Risk of Composite Renal Outcome

Supplement 2. Data Sharing Statement
