# Supplementary material for: Nocturnal Stage 1 Hypertension Defined by 2025 Guidelines in Adults With Chronic Kidney Disease
Source: JAMA Netw Open. 2026 Jan 14;9(1):e2554035. doi: 10.1001/jamanetworkopen.2025.54035 (PMC12805449; doi:10.1001/jamanetworkopen.2025.54035)
Supplement: Supplement 1. — eTable 1. Baseline Characteristics in the Matched Cohorts eTable 2. The Association Between Blood Pressure as Continuous Variables and Primary and Secondary Outcomes eTable 3. Association Between Nocturnal Hypertension and Composite Renal Outcome Among Younger and Older Groups eFigure 1. Flow Chart eFigure 2. Propensity Score Matching for Stage 1 vs Nonhypertension, Stage 2 vs Nonhypertension Cohorts eFigure 3. Restricted Cubic Spline Curves of Nocturnal SBP/DBP With Composite Renal Outcome eFigure 4. Competing Risk Model With Mortality as the Competing Risk, Showing Risk of Composite Renal Outcome With Stage 1 Hypertension eFigure 5. Association Between Nocturnal Blood Pressure Phenotype With (A) Composite Kidney Outcome, (B) WRF and (C) KFRT eFigure 6. Forest Plots for the Subgroup Analyses of the Association Between Nocturnal BP Categories and the Risk of Composite Renal Outcome [file jamanetwopen-e2554035-s001.pdf]

Supplemental Online Content

Zhang T, Zhou Z, Li Q, et al. Nocturnal stage 1 hypertension defined by 2025 guidelines in adults with chronic kidney disease. *JAMA Netw Open*. 2026;9(1):e2554035. doi:10.1001/jamanetworkopen.2025.54035

eTable 1. Baseline Characteristics in the Matched Cohorts
eTable 2. The Association Between Blood Pressure as Continuous Variables and Primary and Secondary Outcomes
eTable 3. Association Between Nocturnal Hypertension and Composite Renal Outcome Among Younger and Older Groups
eFigure 1. Flow Chart
eFigure 2. Propensity Score Matching for Stage 1 vs Nonhypertension, Stage 2 vs Nonhypertension Cohorts
eFigure 3. Restricted Cubic Spline Curves of Nocturnal SBP/DBP With Composite Renal Outcome
eFigure 4. Competing Risk Model With Mortality as the Competing Risk, Showing Risk of Composite Renal Outcome With Stage 1 Hypertension
eFigure 5. Association Between Nocturnal Blood Pressure Phenotype With (A) Composite Kidney Outcome, (B) WRF and (C) KFRT
eFigure 6. Forest Plots for the Subgroup Analyses of the Association Between Nocturnal BP Categories and the Risk of Composite Renal Outcome

This supplemental material has been provided by the authors to give readers additional information about their work.

© 2026 Zhang T et al. *JAMA Network Open*.

eTable 1. Baseline Characteristics in the Matched Cohorts

Variables	Cohort 1			Cohort 2		
	Non-HTN n =292	Stage 1 HTN n =292	*P*-value	Non-HTN n =363	Stage 1 HTN n =363	*P*-value
Age, years	40.2 ± 14.1	40.9 ± 14.4	0.481	39.0 ± 13.0	39.8 ± 13.6	0.442
Male, *n* (%)	158 (54.1)	154 (52.7)	0.803	172 (47.4)	172 (47.4)	0.999
BMI, kg/m^2	23.3 ± 3.8	23.2 ± 3.4	0.840	22.9 ± 3.3	22.9 ± 3.7	0.790
Cigarette smoking, *n* (%)	64 (21.9)	57 (19.5)	0.540	77 (21.2)	75 (20.7)	0.927
Diabetes mellitus, *n* (%)	27 (9.2)	28 (9.6)	0.999	31 (8.5)	28 (7.7)	0.786
CVD history, *n* (%)	6 (2.1)	7 (2.4)	0.999	2 (0.6)	7 (1.9)	0.180
Antihypertensive drugs, *n* (%)	163 (55.8)	158 (54.1)	0.739	200 (55.1)	199 (54.8)	0.999
Hemoglobin, g/dL	13.1 ± 2.1	13.2 ± 2.0	0.648	13.0 ± 2.2	13.0 ± 2.0	0.949
Albumin, g/dL	3.7 ± 0.9	3.8 ± 0.8	0.299	3.6 ± 0.9	3.6 ± 0.9	0.840
HDL-C, mg/dL	46.4 ± 15.5	46.4 ± 15.5	0.835	50.3 ± 19.3	50.3 ± 19.3	0.508
Serum creatinine, mg/dL	0.9 (0.7-1.1)	1.0 (0.7-1.2)	0.741	0.9 (0.7-1.2)	0.9 (0.7-1.2)	0.752
UPCR, mg/g	183.2 (40.2-669.3)	177.1 (56.1-620.0)	0.628	310.2 (73.4-846.9)	161.0 (41.6-646.8)	0.001

Data are presented as mean ± SD, median (IQR), or numbers (percentage). BMI indicates body mass index; CVD, cardiocerebrovascular disease; HDL-C, high-density lipoprotein cholesterol; HTN, hypertension; UPCR, urine protein-to-creatinine ratio.

© 2026 Zhang T et al. *JAMA Network Open*.

eTable 2. The Association Between Blood Pressure as Continuous Variables and Primary and Secondary Outcomes

	Composite renal outcome		WRF		KFRT	
**Variables**	**HR (95% CI)**	***P* value**	**HR (95% CI)**	***P* value**	**HR (95% CI)**	***P* value**
Nocturnal blood pressure per 10 mmHg						
SBP	1.10 (0.98-1.23)	0.094	1.03 (0.88-1.20)	0.705	1.14 (0.98-1.32)	0.097
DBP	1.13 (0.97-1.33)	0.123	1.14 (0.92-1.41)	0.238	1.06 (0.86-1.32)	0.590
Nocturnal blood pressure per 1 SD						
SBP	1.19 (0.97-1.45)	0.094	1.05 (0.80-1.39)	0.705	1.26 (0.96-1.64)	0.097
DBP	1.17 (0.96-1.42)	0.123	1.17 (0.90-1.53)	0.238	1.08 (0.82-1.41)	0.590

Multivariable Cox analysis was adjusted for age, sex, BMI, cigarette smoking, diabetes mellitus, CVD history, antihypertensive drugs, hemoglobin, albumin, HDL-C, serum creatinine, UPCR and daytime SBP/DBP. BMI, body mass index; CVD, cardiocerebrovascular disease; DBP, diastolic blood pressure; HDL-C, high-density lipoprotein cholesterol; KFRT, kidney failure requiring replacement therapy; SBP, systolic blood pressure; UPCR, urine protein-to-creatinine ratio; WRF, worsening renal function.

© 2026 Zhang T et al. *JAMA Network Open.*

eTable 3. Association Between Nocturnal Hypertension and Composite Renal Outcome Among Younger and Older Groups

	Composite renal outcome			WRF		
**Nocturnal BP**	**Event/Total**	**HR (95% CI)**	***P*-value**	**Event/Total**	**HR (95% CI)**	***P*-value**
**Age ≥65 years**						
Non-hypertension	4/20	5.50 (1.05-28.71)	0.043	4/20	5.65 (1.02-31.32)	0.047
Stage 1 hypertension	3/34	Ref.	Ref.	3/34	Ref.	Ref.
Stage 2 hypertension	35/176	1.82 (0.49-6.82)	0.375	21/176	1.50 (0.67-5.89)	0.560
**Age <65 years**						
Non-hypertension	10/416	0.29 (0.14-0.60)	<0.001	8/416	0.29 (0.13-0.66)	0.003
Stage 1 hypertension	27/311	Ref.	Ref.	20/311	Ref.	Ref.
Stage 2 hypertension	256/1461	0.93 (0.61-1.44)	0.752	130/1461	1.03 (0.62-1.73)	0.908

Multivariable Cox analysis was adjusted for sex, BMI, cigarette smoking, diabetes mellitus, CVD history, antihypertensive drugs, hemoglobin, serum creatinine, UPCR and daytime SBP. BP blood pressure; CVD, cardiocerebrovascular disease; SBP, systolic blood pressure; UPCR, urine protein-to-creatinine ratio; WRF, worsening renal function.

© 2026 Zhang T et al. *JAMA Network Open*.

eFigure 1. Flow Chart

ABPM, ambulatory blood pressure monitoring; CKD, chronic kidney disease.

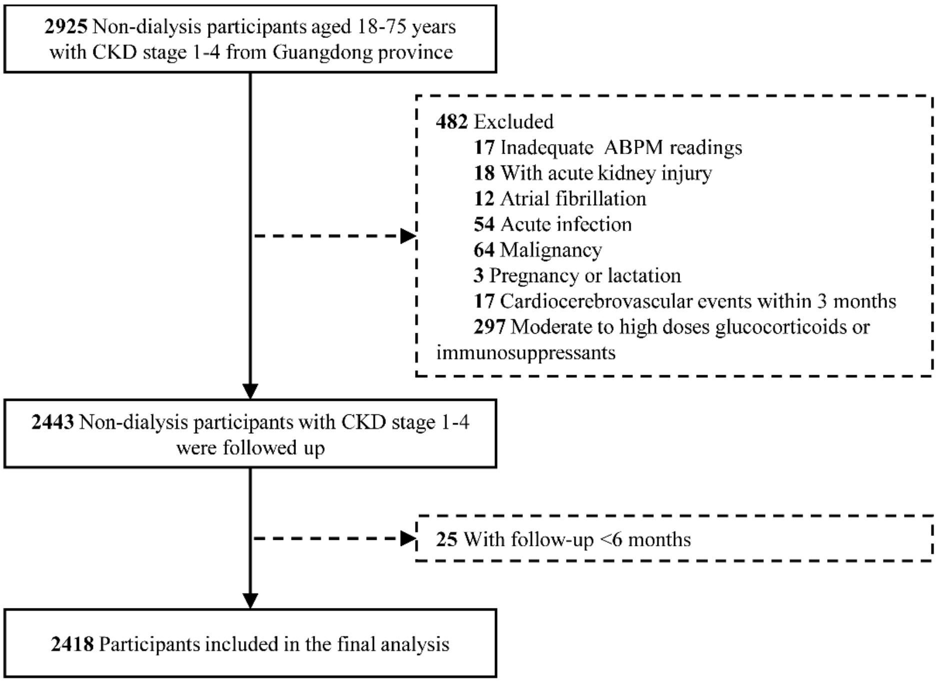

© 2026 Zhang T et al. *JAMA Network Open.*

eFigure 2. Propensity Score Matching for Stage 1 vs Nonhypertension, Stage 2 vs Nonhypertension Cohorts

(A)The density distribution of propensity score and (B) standard mean difference before and after PSM; (C) Kaplan-Meier survival curves after PSM.

HTN, hypertension; PSM, propensity score matching.

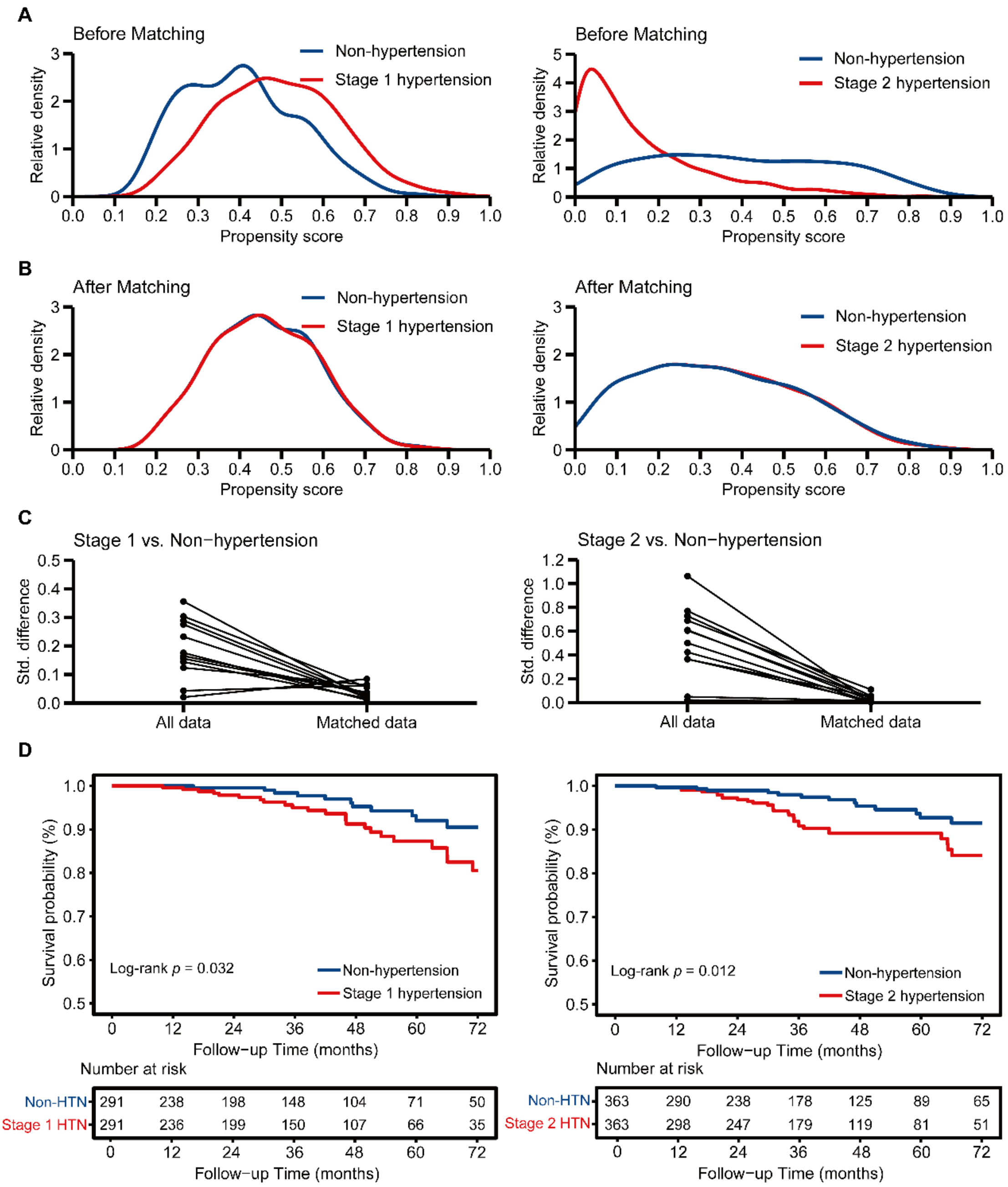

© 2026 Zhang T et al. *JAMA Network Open.*

eFigure 3. Restricted Cubic Spline Curves of Nocturnal SBP/DBP With Composite Renal Outcome

Solid lines indicate HRs, and dashed lines indicate 95% CIs. DBP, diastolic blood pressure; SBP, systolic blood pressure.

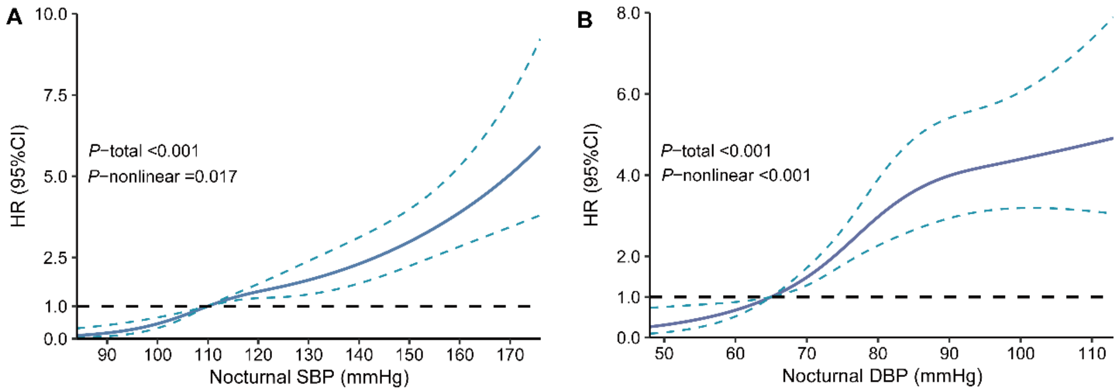

© 2026 Zhang T et al. *JAMA Network Open.*

eFigure 4. Competing Risk Model With Mortality as the Competing Risk, Showing Risk of Composite Renal Outcome With Stage 1 Hypertension

HTN, hypertension.

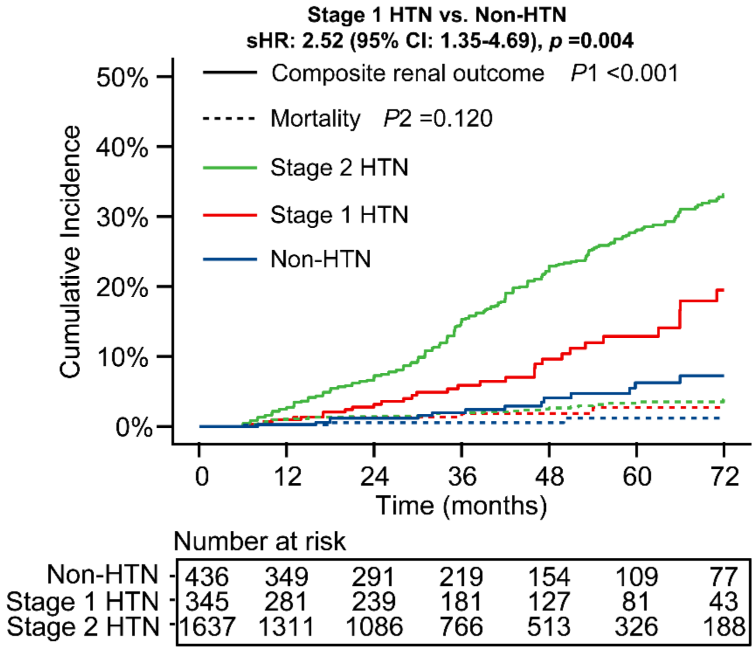

© 2026 Zhang T et al. *JAMA Network Open.*

eFigure 5. Association Between Nocturnal Blood Pressure Phenotype With (A) Composite Kidney Outcome, (B) WRF and (C) KFRT

Model 1: adjusted for age, sex, BMI; Model 2: adjusted for Model 1 plus cigarette smoking, diabetes mellitus, CVD history, antihypertensive drugs, hemoglobin, albumin, HDL-C, serum creatinine and UPCR; Model 3: adjusted for model 2 plus daytime SBP. BMI, body mass index; CVD, cardiocerebrovascular disease; HDL-C, high-density lipoprotein cholesterol; KFRT, kidney failure requiring replacement therapy; SBP, systolic blood pressure; UPCR, urine protein-to-creatinine ratio. WRF, worsening renal function.

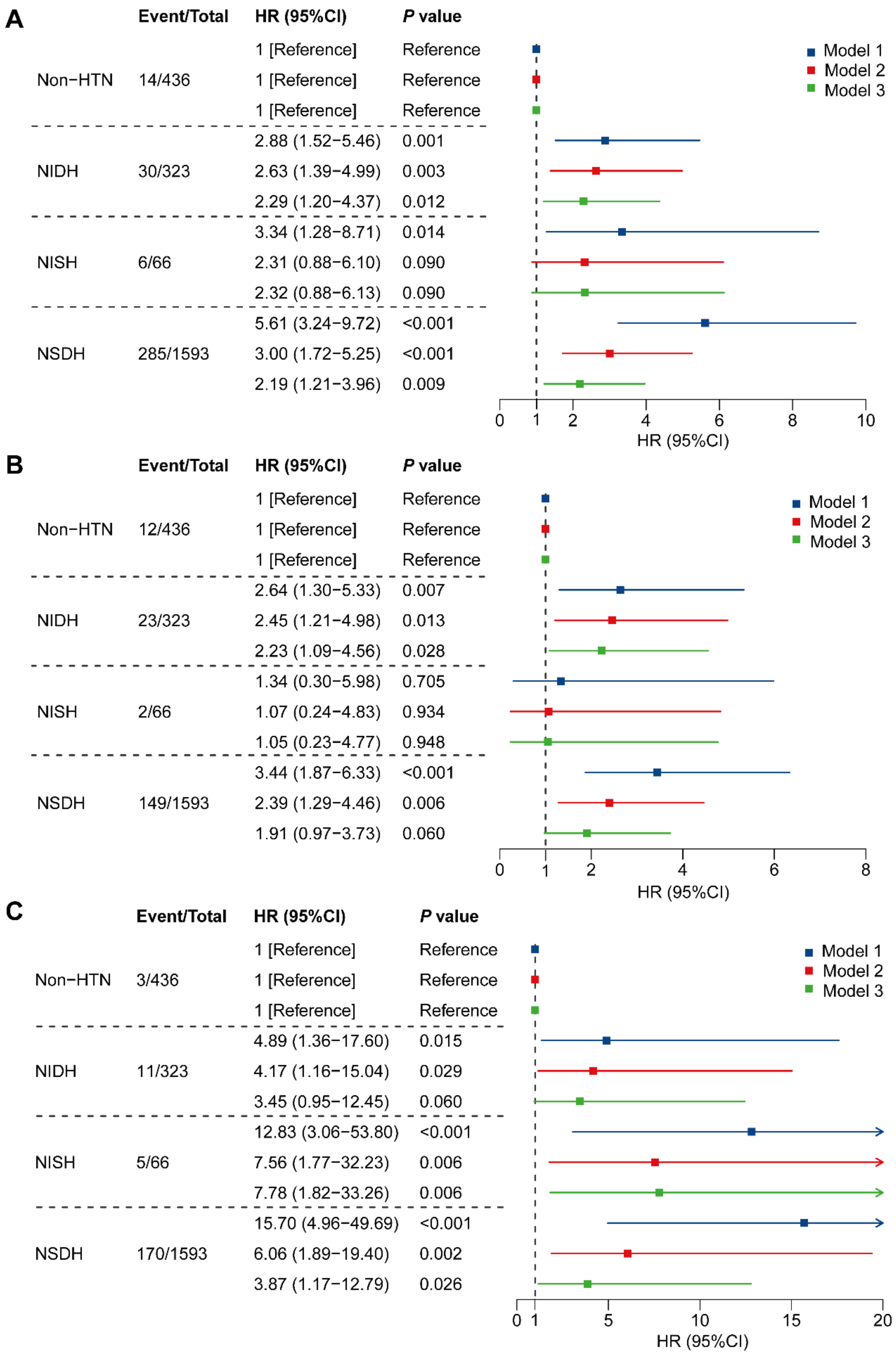

© 2026 Zhang T et al. *JAMA Network Open.*

eFigure 6. Forest Plots for the Subgroup Analyses of the Association Between Nocturnal BP Categories and the Risk of Composite Renal Outcome

BP, blood pressure; CVD, cardiocerebrovascular disease; eGFR, estimated glomerular filtration rate; HTN, hypertension.

© 2026 Zhang T et al. *JAMA Network Open*.

Subgroup	Nocturnal BP	Event/Total	HR (95%CI)	*P* value	*P* for interaction
All people					
	Non-HTN	14/436	reference	reference	
	Stage 1 HTN	30/345	2.49(1.31-4.72)	0.005	
	Stage 2 HTN	291/1637	2.48 (1.38-4.44)	0.002	
Sex					0.080
Female					
	Non-HTN	4/252	reference	reference	
	Stage 1 HTN	12/150	3.24 (1.03-10.17)	0.044	
	Stage 2 HTN	105/604	3.07 (1.05-9.01)	0.041	
Male					
	Non-HTN	10/184	reference	reference	
	Stage 1 HTN	18/195	2.08 (0.95-4.56)	0.068	
	Stage 2 HTN	186/1033	1.92 (0.95-3.85)	0.068	
eGFR					0.070
<60					
	Non-HTN	10/67	reference	reference	
	Stage 1 HTN	18/77	1.46 (0.67-3.21)	0.343	
	Stage 2 HTN	230/755	1.23 (0.62-2.44)	0.556	
≥60					
	Non-HTN	4/369	reference	reference	
	Stage 1 HTN	12/268	4.50 (1.43-14.15)	0.010	
	Stage 2 HTN	61/882	6.34 (2.11-19.07)	0.001	
UPCR					0.671
<500					
	Non-HTN	7/313	reference	reference	
	Stage 1 HTN	17/236	3.08 (1.26-7.49)	0.013	
	Stage 2 HTN	88/863	2.21 (0.93-5.26)	0.072	
≥500					
	Non-HTN	7/123	reference	reference	
	Stage 1 HTN	13/109	2.24 (0.88-5.68)	0.089	
	Stage 2 HTN	203/774	2.53 (1.13-5.67)	0.024	
CVDhistory					0.271
No					
	Non-HTN	12/429	reference	reference	
	Stage 1 HTN	29/329	2.86 (1.45-5.63)	0.002	
	Stage 2 HTN	252/1469	2.87 (1.54-5.38)	0.001	
Yes					
	Non-HTN	2/7	reference	reference	
	Stage 1 HTN	1/16	1.35 (0.09-19.80)	0.829	
	Stage 2 HTN	39/168	1.17 (0.18-7.59)	0.868	
Diabetes mellitus					0.318
NO					
	Non-HTN	11/408	reference	reference	
	Stage 1 HTN	28/303	2.85 (1.41-5.75)	0.004	
	Stage 2 HTN	192/1240	2.61 (1.34-5.08)	0.005	
Yes					
	Non-HTN	3/28	reference	reference	
	Stage 1 HTN	2/42	1.24 (0.20-7.86)	0.819	
	Stage 2 HTN	99/397	1.69 (0.48-5.92)	0.413	
History of Hypertension					0.730
No					
	Non-HTN	1/347	reference	reference	
	Stage 1 HTN	3/211	3.47 (1.44-8.34)	0.006	
	Stage 2 HTN	17/417	4.20 (1.72-10.25)	0.002	
Yes					
	Non-HTN	2/89	reference	reference	
	Stage 1 HTN	7/134	1.78 (0.68-4.64)	0.241	
	Stage 2 HTN	159/1220	1.65 (0.71-3.81)	0.241	
Antihypertensive Drugs					0.926
No					
	Non-HTN	5/218	reference	reference	
	Stage 1 HTN	10/146	2.48 (0.82-7.47)	0.107	
	Stage 2 HTN	37/409	2.09 (0.72-6.09)	0.177	
Yes					
	Non-HTN	9/218	reference	reference	
	Stage 1 HTN	20/199	2.34 (1.06-5.18)	0.035	
	Stage 2 HTN	254/1228	2.42 (1.19-4.93)	0.015	

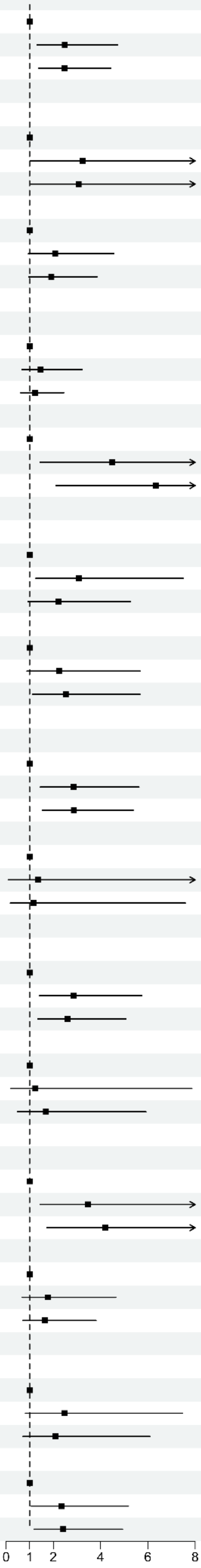

© 2026 Zhang T et al. *JAMA Network Open*.
