# Supplementary material for: Nocturnal Stage 1 Hypertension Defined by 2025 Guidelines in Adults With Chronic Kidney Disease
Source: JAMA Netw Open. 2026 Jan 14;9(1):e2554035. doi: 10.1001/jamanetworkopen.2025.54035 (PMC12805449; doi:10.1001/jamanetworkopen.2025.54035)
Supplement: Supplement 2. — Data Sharing Statement [file jamanetwopen-e2554035-s002.pdf]

Data Sharing Statement

Zhang. Nocturnal Stage 1 Hypertension Defined by 2025 Guidelines in Adults With Chronic Kidney Disease. *JAMA Netw Open*. Published January 14, 2026.
doi:10.1001/jamanetworkopen.2025.54035

Data
Data available: No
